# Prevalence and characteristics of suicide attempters and ideators among acutely admitted psychiatric hospital patients in northwest Russia and northern Norway

**DOI:** 10.1186/s12888-015-0545-3

**Published:** 2015-08-04

**Authors:** Tore Sørlie, Knut W Sørgaard, Anatoly Bogdanov, Trond Bratlid, Grigory Rezvy

**Affiliations:** 1 Institute of Clinical Medicine, University of Tromsø, 9037 Tromsø, Norway; 2 Department of General Psychiatry, University Hospital of North Norway, Tromsø, Norway; 3 Nordland Hospital Trust, 8092 Bodø, Norway; 4 Archangelsk Clinical Psychiatric Hospital, Archangelsk, Russia; 5 North State Medical University, Archangels, Russia

**Keywords:** Acute psychiatric patients, Suicide risk profile, Transcultural study

## Abstract

**Background:**

More knowledge about suicidality and suicide risk profiles in acute psychiatric hospital patients (both first-time and chronic patients) is needed. While numerous factors are associated with suicidality in such populations, these may differ across cultures. Better understanding of factors underlying suicide risk can be informed by cross-cultural studies, and can aid development of therapeutic and preventive measures.

**Methods:**

An explorative, cross-sectional cohort study was carried out. Acutely admitted patients at one psychiatric hospital in northwest Russia and two in northern Norway were included. At admission, demographic, clinical, and service use data were collected, in addition to an assessment of suicidal ideation and attempts, comprising five dichotomic questions. Data from 358 Norwegian and 465 Russian patients were analyzed with univariate and multivariate statistics. Within each cohort, attempters and ideators were compared with patients not reporting any suicidality.

**Results:**

The observed prevalence of suicidal ideation and attempts was significantly higher in the Norwegian cohort than in the Russian cohort (χ^2^ = 168.1, *p* < 0,001). Norwegian suicidal ideators and attempters had more depressed moods, more personality disorders, and greater problems with alcohol/drugs, but fewer psychotic disorders, cognitive problems or overactivity than non-suicidal patients. Russian suicidal ideators and attempters were younger, more often unemployed, had more depressed mood and adjustment disorders, but had fewer psychotic disorders and less alcohol/drug use than the non-suicidal patients.

**Conclusions:**

Rates of suicidal ideation and non-fatal attempts in Norwegian patients were intermediate between those previously reported for patients admitted for the first time and those typical of chronic patients. However, the significantly lower rates of suicidal ideation and non-fatal attempts in our Russian cohort as compared with the Norwegian, contrasted with what might be expected in a region with much higher suicide rates than in northern Norway. We suggest that suicide-related stigma in Russia may reduce both patient reporting and clinicians’ recognition of suicidality. In both cohorts, overlapping risk profiles of ideators and attempters may indicate that ideators should be carefully evaluated and monitored, particularly those with depressed moods, alcohol/substance abuse disorders, and inadequate treatment continuity.

## Background

Suicidality (suicidal ideation and attempts) is a major public health problem worldwide [1], with great variation in rates between culturally diverse sites [2]. The highest suicide rates have been reported in Eastern Europe [3] and in Asian countries, particularly South Korea [4], while the lowest rates have been found in Muslim and Latin American countries [3]. Religiosity can potentially serve as a protective factor against suicidal behavior [5]. In 2009 in Norway, there was a rate of 11.9 (per 100,000 per year) (17.3 for men and 6.5 for women), whereas in 2006 in Russia, a higher rate of 30.1 (53.9 for men and 9.5 for women) was reported [6].

Strong evidence exists that the majority of suicide completers were suffering at the time of death from a psychiatric disorder, most commonly affective disorders and substance use disorders [7]. Reported lifetime risk of suicide is as high as 15 % for those with affective disorders, 10 % for those with schizophrenia, and 2–3 % for alcohol abusers [8]. For patients admitted to psychiatric hospitals, the suicide risk is even higher [9, 10], particularly on first admission [11]. In a large prospective transcultural follow-up study it was demonstrated that early awareness of the disorder in schizophrenia increased the suicidal risk whereas increased awareness due to treatment appeared to be protective [12]. Men who complete suicide are more commonly diagnosed with substance-related problems, personality disorders, and childhood disorders, whereas women are more likely to have an affective disorder [7]. Mood disorders are a common suicide risk factor in high income countries, whereas impulse-control disorders (such as those related to alcohol abuse) may be more significant in low and middle income countries [13]. However, depressed suicide attempters from New York made attempts of greater lethality and reported more lifetime aggressive behavior that depressed attempters in Madrid [14].

Despite all these variables being associated with suicidal behavior, their usefulness in predicting future suicidal behavior still remains unsolved [15]. A recent review of predictors of repeated suicide attempts concluded that the strongest predictor for nonfatal repetitions is a history of suicide attempt [16]. Other significant factors included being a victim of a sexual abuse, poor global functioning, having a psychiatric disorder, undergoing psychiatric treatment, and alcohol abuse or dependence. Completed suicide was most strongly predicted by older age, high levels of suicidal ideation, and a history of suicide attempt. Living alone, being male, and alcohol abuse were weaker predictors. In a Spanish-French cross-sectional study, the risk of frequent suicide attempts was highest among middle-aged subjects, and diminished progressively with advancing age of onset at first attempt. Anxiety disorders significantly increased the risk of presenting frequent suicide attempts [17]. Other studies have emphasized the significance of low education and unemployment [18, 19], as well as the availability of health services [20].

From a clinical perspective, it is particularly important to identify predictors of suicidal ideation and attempt among patients acutely admitted to psychiatric hospitals as their suicide risk is particularly high. A recent comparative study of 168 first-time hospital admitted psychiatric patients in northern Norway found no sociodemographic or clinical differences in risk profiles for suicidal ideation (reported by half the patients) and attempt (reported by one fifth of patients) [21]. However, first-time admissions constitute only a limited proportion of admissions to acute psychiatric wards and more knowledge regarding the risk profiles of all acutely admitted psychiatric patients is needed. Thus, in the current study we investigate representative samples of acute psychiatric patients. Our use of a cross-cultural study spanning two regions with vast differences in suicidal rates, socio-cultural environment, and health service organization ensures greater variability of variables of interest. We also include patients with no suicidality to compare with patients with suicidal ideation or attempted suicide, to explore factors associated with suicidality.

The samples were drawn from northern Norway and Archangelsk, Russia. Both regions are vast, mainly rural areas with a low population density. Rural areas, such as these, are reported to have higher suicide rates than do urban areas [22, 23]. Although suicide rates in northern Norway are similar to rates elsewhere in the country, the 2006 rate in Archangelsk was much higher than the rest of Russia (46.7 versus 30.1) [24]. There are differences in the mental health care provision in each of these contexts. In Norway, mental health care is provided jointly by primary health care and specialized psychiatric services. Conversely, in Russia, mental health care is provided solely by specialized health services, mainly located in hospitals [25]. Furthermore, until recently Russian GPs have neither been expected to diagnose nor to treat mental disorders. In Norway, hospital stays tend to be short with rapid return to the patients’ home, where care continues in close collaboration with primary health services. Russian municipalities have less developed social and health care facilities, demanding greater levels of self-management. Thus, Russian patients often experience longer hospital stays to reach necessary levels of recovery and functioning [26].

The aims of this study were to identify the prevalence and predictors of suicidal ideation and attempts in acute psychiatric patients, prior to a hospital stay, in northern Norway and Archangelsk. We hypothesized that the rates of suicidal ideation and attempts among our mixed sample representing all inpatients would be lower than previously reported for first-time hospital admitted patients, but higher than usually found in chronic patients. We assumed that greater suicide-related stigma in Russia would reduce reported suicidality in the Archangels region below what might be expected from the very high regional suicide figures.

## Methods

### Procedures

#### Participants

Participants were included from three hospitals: Archangelsk Clinical Psychiatric Hospital, Russia (seven acute wards with 900 emergency psychiatric beds), Nordland County Psychiatric Hospital, Norway (two acute wards with 100 emergency psychiatric beds), and University Hospital of North Norway (three acute wards with 147 emergency psychiatric beds). Further details regarding the study contexts have been published elsewhere [26]. All admissions to these wards were included for three months in 2005 in Norway, and between 2008 and 2009 in Russia. Data were included only for the participant’s first admission during the study period. A total of 841 admissions were analyzed (377 Norwegian, 464 Russian).

#### Assessments

Data were collected during clinical consultations by the patients’ therapists (psychiatrists or psychologists). Therapists responsible for the assessments were systematically trained and details of the training procedure are published elsewhere [26].

A form developed for use in a national Norwegian acute ward study [27] was administered to collect data regarding the referral and admission, patient demographics, services received before admission, and assessments made at admission. The HoNOS [28, 29] and the Global Assessment of Functioning (GAF) [30] are integrated within this form. The HoNOS, which was developed at the UK Royal College of psychiatrists as a routine outcome measure in mental health services, comprises four subscales, which include behaviors (aggression/disruptive behavior, self-harm, alcohol use, and substance use), impairment (cognition and physical health), symptoms (hallucinations and delusions, depression and other symptoms), and social function (social relations, general functioning, housing situation, and activities). In addition, ICD-10 diagnoses as rated by clinicians were recorded.

At admission and as part of the HoNOS assessment, the presence of suicidal ideation or attempts was rated on the following dichotomous questions (rated ‘present’/‘not present’): (a) no suicidal thoughts or plans, (b) passive wishes to be dead, (c) thoughts of suicide, (d) planning suicide, and (e) lifetime attempted suicide. The group of suicidal ideators comprised those who gained a positive score (i.e. assessed as ‘present’) on either b, c or d.

### Statistical analyses

#### Predictor variables

Informed by the literature, several variables were investigated as potential predictors of suicidal ideation and attempts. These included the following demographic variables: gender, age (<40 years or ≥ 40 years), living circumstances (alone versus cohabitation), education, and employment status. Clinical variables investigated were the HoNOS (dichotomized as problem present or not), ICD-10 diagnoses (psychoses, affective disorders, alcohol and substance use disorders, personality disorders, adjustment disorder), and GAF symptom and functioning scores (<40 or ≥ 40). We also assessed the effects of previous psychiatric treatment (whether or not received and whether in the last year), and the circumstances of the current admission (whether the patient had agreed with admission or not, and whether they had been referred to hospital by someone who knows them or not).

#### Statistics

Missing data was < 6 % on all recorded variables, and no substitutions were made. In addition, given that the sample size was large enough to allow for the number of predictor variables used, no correction for multiple testing was necessary. All independent variables were dichotomized to avoid obtaining small numbers of observations within cells in the regression analyses. Demographic, clinical, and health service use data were analyzed using the chi-square test.

Logistic regressions (univariate and multivariate) were used to identify predictors of suicide attempters and ideation, with reference to the group with no reported suicidality. Each cohort (Norway and Russia) was analyzed separately. Except for high correlations between GAF symptom and functioning scores in both cohorts (analyzed using Spearman’s rho), all correlations between the predictor variables were small to moderate. Potential explanatory variables were chosen with a significance value of .25 on univariate analyses as criteria for inclusion [31]. Initially, sociodemographic variables were entered. Clinical and then health service-related variables were subsequently added. Variables that were not multivariately significant (*p* ≤ .05), either with suicide attempts or ideation during the stepwise regression analyses, were removed, and subsequent analyses were run without them [32]. Odds ratios (OR) were used to indicate the effect sizes of the predictors and Nagelkerke R-square was used to estimate the explanatory power of the regression models.

### Ethics

The regional ethical committees in Northern Norway and at the Medical University in Archangelsk approved the study. It was agreed that patients unable to give informed consent could be included to obtain a representative patient sample. In Russia, each patient’s participation in the study had to be recommended by their therapists.

## Results

### Sample characteristics

#### Demographics and healthcare use in the Norwegian and Russian patients

There was no difference in age between the Norwegian (mean [M] = 46.47, SD = 15.40, range = 25–93) and Russian cohorts (M = 46.53, SD = 14.07, range = 21–85). The Russian patients were more often male, married, cohabiting, and either working or a student. More Russian patients agreed with their admission and they had less often received previous psychiatric treatment (72 % of Russians and 83 % of Norwegians). Additionally, the Norwegian patients were more often receiving disability pension.

#### Clinical characteristics of Norwegian and Russian patients

Some clinical differences between the cohorts were observed. Norwegians were less often evaluated as overactive and less often had problems with alcohol/drugs. More Russian than Norwegian patients had GAF-S and GAF-F scores greater than 40. Russians were also more often diagnosed with alcohol/drug-related disorders and adjustment disorders. In contrast, the Norwegians were more often diagnosed with affective disorders, psychosis and personality disorders (Table 1).

**Table 1:** Sociodemographic, clinical, and service use characteristics in Norwegian and Russian acute psychiatric patients

Variable	Norwegian cohort (*n* = 377) n (%)	Russian cohort (*n* = 464) n (%)	χ^2^
Age < 40	141 (37.5)	160 (34.6)	0.74
Male	194 (51.6)	284 (63.4)	7.62**
Unmarried	288 (76.4)	257 (55.3)	40.69***
Living alone	202 (55.6)	124 (26.6)	128.28***
Working/student	41 (10.9)	117 (25.2)	27.88***
Disability pension	160 (45.2)	142 (30.6)	20.35***
Overactive (HoNOS 1)	200 (53.1)	294 (63.4)	9.13**
Alcohol/drug problems (HoNOS 3)	130 (34.9)	273 (59.5)	49.50***
Cognitive problems (HoNOS 4)	163 (44.6)	230 (49.8)	2.14
Depressed moods (HoNOS 7)	263 (71.7)	357 (76.1)	3.01
Problems with activities of daily life (HoNOS 10)	242 (66.1)	305 (65.9)	0.01
GAF-S ≥ 40	134 (36.7)	208 (44.7)	5.52*
GAF-F ≥ 40	146 (40.0)	217 (46.7)	3.80*
Affective disorder	99 (26.3)	17 (3.7)	89.55***
Psychosis	127 (33.7)	110 (23.7)	10.36**
Alcohol/drug disorder	33 (8.8)	217 (46.7)	143.36***
Personality disorder	20 (5.3)	3 (0.6)	17.00***
Adjustment disorder	12 (3.2)	18 (3.9)	0.29
Previous lifetime psychiatric treatment	298 (83.4)	336 (72.0)	14.02**
Psychiatric treatment in last year	181 (48.1)	256 (55.0)	2.37
Referred by someone who knows patient	180 (48.1)	192 (42.8)	2.37
Wanted to be admitted	209 (59.4)	332 (69.4)	17.4***

*p ≤ 0.05, ** *p* < 0.01, *** *p* < 0.001

More detailed description of characteristics of the patients in the study sample is published elsewhere [24].

### Suicidal ideation and attempts

#### Prevalence of suicidal ideation and attempts in Norwegian and Russian cohorts

The prevalence of suicidality was significantly higher in the Norwegian cohort than in the Russian cohort (χ^2^ = 168.1, *p* < 0,001). Suicidal ideation was reported by 134 (36.8 %) of the Norwegian patients and 30 (7.1 %) of the Russians patients prior to their admission. Among the Norwegian patients, 50 (13.8 %) had attempted suicide. In the Russian cohort, 22 (5.2 %) had attempted suicide. In the Russian cohort, more women than men had suicidal ideation, whereas there were no sex-related differences in the Norwegian cohort (Table 2).

**Table 2:** Sex-related suicidality of psychiatric patients prior to hospital admission in Northern Norway and Archangelsk

	Norwegian cohort			Russian cohort			Total study sample		
	Men n (%)	Women n (%)	χ^2^	Men n (%)	Women n (%)	χ^2^	Men n (%)	Women n (%)	χ^2^
No suicidal thoughts or plans	93 (50.5)	85 (47.8)	0.29 *p* = 0.87	230 (90.2)	142 (84.0)	7.45 *p* = 0.024	178 (49.2)	372 (87.8)	168.1 *p* < 0.001
Suicidal ideators	66 (35.9)	68 (38.2)		11 (4.3)	19 (11.3)		134 (37.0)	30 (7.0)	
Suicidal attempters	25 (13.6)	25 (14.0)		14 (5.5)	8 (4.7)		50 (13.8)	22 (5.2)	
Total	184 (100)	178 (100)		255 (100)	169 (100)		362 (100)	424 (100)	

#### Predictors of suicidal ideation and attempts in the Norwegian cohort

- Univariate analyses

Using univariate methods, patients without any suicidality were compared with suicide attempters. Attempters were younger than the other group, with more patients less than 40 years old (OR = 1.89 [95 % CI 1.00–3.59], *p* < 0.05). They were also less often referred by someone who knew them than were patients without any suicidality (OR = 0.43 [95 % CI 0.22–0.84], *p* < 0.05). Attempters less often had problems with activities of daily life (OR = 0.37 [95 % CI 0.19–0.71], *p* < 0.01) and were more often employed (OR = 2.72 [95 % CI 1.14–6.50], *p* < 0.05). Clinically, attempters had more affective disorders than patients without any suicidality (OR = 2.23 [95 % CI 1.10–4.54], *p* < 0.05).

Comparing suicide ideators with patients with no suicidality, we found that the former were more often younger than 40 years (OR = 1.57 [95 % CI 0.99–2.51], *p* < 0.05). They had less often been referred by someone who knew them (OR = 0.63 [95 % CI 0.40–0.99], *p* < 0.05) and had fewer problems with activities of daily life (OR = 0.60 [95 % CI 0.37–0.98], *p* < 0.05). Similarly to attempters, ideators had more affective disorders than patients without any suicidality (OR = 2.79 [95 % CI 1.66–4.70], *p* < 0.001).

b) Multivariate analyses

Our final model explained 43 % of the variance in suicidal ideation and attempts (Nagelkerke R-square).

Compared with those not reporting any suicidality, both attempters (OR = 14.13 [95 % CI 3.96–50.41], *p* < 0.001) and ideators (OR = 7.69 [95 % CI 3.74–15.80], *p* < 0.001) more often reported depressed moods. There was also a higher rate of personality disorder in both attempters (OR = 3.93 [95 % CI 0.61–25.23]) and ideators (OR = 6.74 [95 % CI 1.54–29.44], *p* < 0.05) than in those not reporting suicidality. More attempters (OR = 2.68 [95 % CI 1.21–5.94], *p* < 0.05) and ideators (OR = 2.19 [95 % CI 1.21–3.95], *p* < 0.05) had GAF-F scores ≥ 40. There was also a greater rate of alcohol/drug problems in both attempters (OR = 2.83 [95 % CI 1.30–6.18], *p* < 0.01) and ideators (OR = 1.63 [95 % CI 0.89–2.98]) than in patients without suicidality.

Patients with suicidal ideation and attempts showed fewer problems in some clinical areas than did patients without any suicidality. Both attempters (OR = 0.23 [95 % CI 0.10–0.55], *p* < 0.01) and ideators (OR = 0.64 [95 % CI 0.36–1.13]) had fewer cognitive problems than patients without suicidality. There were also fewer problems with overactivity in attempters (OR = 0.91 [95 % CI 0.42–2.00], *p* < 0.05) and ideators (OR = 0.48 [95 % CI 0.27–0.86], *p* < 0.05) compared with the other patient group. Finally, both attempters (OR = 0.53 [95 % CI 0.22–1.28], *p* < 0.01) and ideators (OR = 0.27 [95 % CI 0.14–0.51], *p* < 0.001) had fewer psychotic diagnoses than patients without suicidality.

#### Predictors of suicidal ideation and attempts in the Russian cohort

- Univariate analyses

Univariate analysis to compare those not reporting any suicidality with suicide attempters showed that attempters less often reported problems with activities of daily life (OR = 0.34 [95 % CI 0.14–0.82], *p* < 0.05). Attempters also had fewer alcohol/drug problems (OR = 0.35 [95 % CI 0.13–0.98], *p* < 0.05) and fewer psychotic disorders (OR = 0.27 [95 % CI 0.13–0.58], *p* < 0.05).

Comparing suicide ideators with patients with no suicidality, we found that the former were less often male (OR = 0.36 [95 % CI 0.17–0.77], *p* < 0.01). They also had fewer psychotic disorders (OR = 0.23 [95 % CI 0.13–0.58], *p* < 0.001) and fewer alcohol/drug problems (OR = 0.44 [95 % CI 0.19–1.01], *p* < 0.05). Table 3 shows the results of all univariate and multivariate analyses in both cohorts.

**Table 3:** Suicidal ideation and attempts in psychiatric patients compared with patients not reporting any suicidality

	Russian cohort (95 % CI)				Norwegian cohort (95 % CI)			
	Attempters		Ideators		Attempters		Ideators	
	Univariate	Multivariate	Univariate	Multivariate	Univariate	Multivariate	Univariate	Multivariate
Male sex	1.08 (0.44–2.64)		0.36 (0.17–0.77)**		0.91 (0.49–1.71)		0.89 (0.57–1.39)	
Age < 40	7.26 (2.62–20.15)***	5.25 (1.74–15.86)**	1.24 (0.57–2.68)	1.38 (0.60–3.14)	1.89 (1.00–3.59)*		1.57 (0.99–2.51)*	
Working/student	1.01 (0.83–2.64)	0.42 (0.12–1.39)	0.19 (0.05–0.81)*	0.14 (0.03–0.61)**	2.72 (1.14–6.50)*		1.26 (0.59–2.70)	
Overactive (HoNOS 1)	1.61 (0.62–4.21)		2.41 (0.96–6.05)		0.64 (0.34–1.20)	0.91 (0.42–2.00)	0.37 (0.23–0.59)***	0.48 (0.27–0.86)*
Problems alc/drug (HoNOS 3)	1.54 (0.14–3.87)	1.09 (0.35–3.45)	0.42 (0.19–0.90)*	0.31 (0.13–0.72)**	2.62 (1.37–5.01)**	2.83 (1.30–6.18)**	1.34 (0.83–2.15)	1.63 (0.89–2.98)
Cognitive problems (HoNOS 4)	0.56 (0.23–1.37)		0.85 (0.41–1.81)		0.18 (0.08–0.39)***	0.23 (0.10–0.55)**	0.49 (0.31–0.78)**	0.64 (0.36–1.13)
Depressed moods (HoNOS 7)	2.30 (0.67–7.93)	1.81 (0.43–7.61)	10.52 (1.41–78.23) ***	16.48 (2.18–124.79)**	14.95 (4.48–49.89)***	14.13 (3.96–50.41)***	8.88 (4.15–16.95)***	7.69 (3.74–15.80)***
Problems activities (HoNOS 10)	0.34 (0.14–0.82)*		0.67 (0.39–1.83)		0.37 (0.19–0.71)**		0.60 (0.37–0.98)*	
GAF-S ≥40	1.05 (0.44–2.48)		1.43 (0.68–3.02)		3.38 (1.73–6.63)***		4.42 (2.68–7.30)***	
GAF-F ≥40	0.97 (0.41–2.30)		1.73 (0.82–3.72)		3.75 (1.93–7.29)***	2.68 (1.21–5.94)*	3.03 (1.88–4.90)***	2.19 (1.21–3.95)*
Affective disorder (ICD-10)	1.32 (0.16–10.54)		3.07 (0.82–11.43)		2.23 (1.10–4.54)*		2.79 (1.66–4.70)***	
Psychosis (ICD-10)	0.27 (0.13–0.58)*		0.23 (0.13–0.58)***		0.27 (0.13–0.58)**	0.53 (0.22–1.28)	0.23 (0.13–0.38)***	0.27 (0.14–0.51)***
Personality disorder (ICD-10)					1.84 (0.93–3.64)	3.93 (0.61–25.23)	4.24 (1.34–13.47)*	6.74 (1.54–29.44)*
Adjustment disorder (ICD-10)	31.50 (10.47–94.76)***	24.32 (6.62–89.36) ***	1.57 (0.19–12.98)*	1.73 (0.19–16.13)	11.30 (1.15–111.11)		11.15 (1.38–90.26)*	
Alcohol/drug disorder (ICD-10)	0.35 (0.13–0.98)*		0.44 (0.19–1.01)*		1.73 (0.62–4.81)		1.47 (0.67–3.24)	
Referred by someone who knows patient	0.26 (0.09–0.77)*	0.36 (0.10–1.26)	0.58 (0.26–1.27)*	0.35 (0.15–0.83)*	0.43 (0.22–0.84)*		0.63 (0.40–0.99)*	

Patients with no suicidality used as reference category; Personality disorders got no estimates in the Russian cohort owing to few subjects (*N* = 3); Significance: *p ≤ 0.05, ** *p* < 0.01, *** *p* < 0.001

b) Multivariate analyses

Our final model explained 30 % of the variance in suicidal ideation and attempts (Nagelkerke R-square).

Compared with those not reporting any suicidality, both attempters (OR = 5.25 [95 % CI 1.74–15.86], *p* < 0.01) and ideators (OR = 1.38 [95 % CI 0.60–3.14]) were more often younger than forty years. Attempters (OR = 0.42 [95 % CI 0.12–1.39]) and ideators (OR = 0.14 [95 % CI 0.03–0.61], *p* < 0.01) were less often employed than patients without suicidality. Both attempters (OR = 24.32 [95 % CI 6.62–89.36], *p* < 0.001) and ideators (OR = 1.73 [95 % CI 0.19–16.13]) had more adjustment disorders than did patients without any suicidality. Attempters (OR = 1.81 [95 % CI 0.43–7.61]) and ideators (OR = 16.48 [95 % CI 2.18–124.79], *p* < 0.01) also more often reported depressed moods than the other patient group. However, ideators had fewer alcohol/drug problems (OR = 0.31 [95 % CI 0.13–0.72], *p* < 0.01). Finally, both attempters (OR = 0.36 [95 % CI 0.10–1.26]) and ideators (OR = 0.35 [95 % CI 0.15–0.83], *p* < 0.05) were less often referred by someone who knew them.

## Discussion

In our sample, one in three Norwegian patients and one in fourteen Russians reported suicidal ideation at hospital admission. Furthermore, one in seven Norwegians and one in twenty Russians reported having made a suicide attempt. As expected, the figures for the Norwegian patients were somewhat higher than previously estimated among chronic patients [11]. However, they are lower than previously reported among patients admitted for the first time from the same catchment area, where half of all patients had suicidal ideation and one in five had made a suicide attempt [21]. The significantly lower suicidality rates observed in the Russians cohort contrast with much higher suicide rates reported for Archangelsk elsewhere [6, 22]. Lower rates of affective and psychotic disorders, as well as more previous psychiatric treatment in the Russian cohort, may partly explain the difference.

A further factor for consideration is differences in the stigma associated with suicide and suicide-survivorship internationally, which can extend to family, friends, and therapists of those who attempt or complete suicide [33]. Differences in cultural attitudes towards both suicide and mental health problems may contribute to differences in suicide rates across different societies either owing to avoidance of such behaviors or a tendency to under-report them. A study of Russian attitudes reported high levels of suicide related stigma [34] and in a transcultural study of public opinions about psychiatric disorders in Novosibirsk and Germany, it was found that Russian respondents had a stronger tendency to consider mental disorders as self-inflicted and were more inclined to rely on help resources outside the mental health sector, for example traditional “alternative” treatment methods [35, 36]. Such suicide-related stigma is likely to prevent an individual from acknowledging suicidal ideas, and will lessen the likelihood that one communicates the feelings to others, such as family members or mental health professionals.

We found that amongst Russian suicidal ideators there were more women than men. This is consistent with observations that Russian women are more inclined to respond to difficult life conditions with subjective stress [23]. In contrast, men more often respond by engaging in negative health lifestyles and self-destructive behaviors [23], such as alcohol abuse, rather than verbalizing their feelings [37]. Previous studies have reported a strong tendency to somatization both among Russian men and women, as well as high rates of alcohol dependence in Russian men, particularly in rural regions (up to 70 %) [38]. It is possible that a failure to acknowledge and communicate suicidal ideation is in itself a risk factor for completing suicide.

These observations suggest that mental illness and suicide-related stigma in both the assessed patients and the assessors may reduce the degree to which suicidal ideation is acknowledged and identified. A probable under-communication of suicidality might be reduced by using a more comprehensive assessment [39] than the five dichotomous questions used to assess suicidal ideation and attempts in our study. However, we used these questions as they were integrated in the HoNOS questionnaire, which has been used in various previous studies to assess behaviors, impairment, symptoms, and social functioning of severely ill psychiatric patients [28, 29]. In addition, better access to mental health services may further facilitate recognition, earlier diagnosis, and treatment of mental disorders associated with suicidality. Thus, we would expect the accessibility of such services in primary healthcare in Norway to reduce the impact of mental health problems and lead to lower rates of suicidal ideation or attempts. However, our finding of high levels of suicidality in our Norwegian cohort may reflect regional differences in healthcare provision [20].

The role of psychiatric diagnoses was also explored in our study. As predicted, we found greater rates of depressive moods and affective disorders, but lower rates of psychotic disorders, in suicide attempters and ideators than in patients not reporting any suicidality, in both Norwegian and Russian patients. This is unsurprising as the association between suicidality and affective disorders is well known [13, 40]. However, the expected positive association between alcohol/drugs problems and suicidality was only observed in the Norwegian cohort. In contrast to previous findings [10, 41], a negative relationship between alcohol/drug disorders and both suicide attempts and ideation was observed in both univariate and multivariate analyses in our Russian sample. We surmise that the use of alcohol may have different functions in the two societies. In contrast to Russia, Norway has low unemployment rates, good welfare and social security, relatively low levels of mental health stigma, and lower consumption of and social acceptance of alcohol. As such, in Norway alcohol abuse may serve as a prominent factor in suicide attempts, and may signal the presence of suicidal ideation. In contrast, in Russia alcohol may be used as a way to displace or distract from suicidal ideation, serving as a form of ‘chronic suicide’ as described by Menninger [42].

The protective effect of employment observed in the Russian cohort is in line with previous research showing an inverse association between income and various important outcomes, including psychological stress, substance use disorders, suicidal ideation, and suicide attempts [43]. Higher employment rates among the Russian patients and a sparse public social support system in the Russian federation probably contribute to this association. That unemployment did not predict suicidality in the Norwegian cohort, may relate to the fact that fewer of the Norwegian patients were working and substantially more lived on social security.

Our study has several limitations. Some features of suicidal behavior were not assessed, such as familial history of suicide. However, the study is explorative and important factors have been highlighted, although the included list of variables is not exhaustive. Generalization of the findings is limited by the fact that the study only included patients from the northern areas of Norway and Russia. Nonetheless, the use of a cross-cultural comparison allows us to explore how cultural factors may influence relationships of demographic and clinical factors with suicidality. There were small numbers of Russian ideators and attempters in our sample, so results of the statistical analysis should be interpreted with caution. Finally, Russian and Norwegians clinicians may have used some diagnostic criteria differently, particularly concerning personality and adjustment disorders. This should be borne in mind when considering the influence of mental health disorders on suicidality in each cohort.

## Conclusions

Our study compares factors associated with suicidal ideation and attempts in northern Norway and Archangelsk, Russia. Although expected rates of suicidal ideation and attempts were observed in the Norwegian cohort, rates in the Russian cohort were lower than expected. We suggest this relates to high levels of suicide-related stigma and the use of a brief assessment measure of suicidal ideation and attempts. A broad and indirect approach to this sensitive topic appears advantageous where stigma is prevalent. The effect of alcohol/drug problems on suicidality also appears to differ depending on context. We suggest that differences in the reasons for alcohol/drug use, as well as cultural attitudes to substances may play a role. Furthermore, cultural differences in social factors, such as employment rates and social support available, appear to alter the influence of such factors on suicidality.

Corresponding risk profiles found in suicidal ideators and attempters in each country indicate that ideators, who represent the most prevalent group among acutely admitted psychiatric patients, should be evaluated and treated with great concern. It appears particularly important to secure continuity of care and early identification of suicidal ideation, as well as ensuring early diagnosis of and treatment for depression and substance abuse.
